# Green Extraction Coupled With Spray‐Drying Encapsulation Enhances Stability and In Vitro Bioaccessibility of Phoenix dactylifera Seed Bioactives

**DOI:** 10.1002/fsn3.72488

**Published:** 2026-10-31

**Authors:** Olfa Rebai, Faten Raboudi, Malek Ben Temessek, Rafik Gatri, Sami Fattouch

**Affiliations:** ^1^ Laboratory of EcoChemistry LR21ES02, National Institute of Applied Sciences and Technology (INSAT) University of Carthage Tunis Tunisia; ^2^ Department of Technics, Higher Institute of Animation for Youth and Culture University of Tunis Tunis Tunisia; ^3^ Laboratory of Selective Organic & Heterocyclic Synthesis Biological Activity Evaluation (LR17ES01), Faculty of Science of Tunis University of Tunis El Manar Tunis Tunisia

**Keywords:** date seed, green extraction, high‐pressure autoclave extraction, in vitro bioaccessibility, INFOGEST 2.0, microencapsulation, polyphenols, spray‐drying, storage stability

## Abstract

The valorization of date seed by‐products as sources of bioactive compounds represents a promising strategy for supporting sustainable and circular food systems. This study evaluated ultrasound‐assisted extraction (UAE), high‐pressure autoclave extraction (HPAE), and sequential UAE followed by HPAE for the recovery of bioactive compounds from Tunisian date seeds. The most effective extraction condition was subsequently subjected to spray drying using an alginate–xanthan gum wall matrix. Encapsulated (EP) and non‐encapsulated (NEP) powders were evaluated for physicochemical properties, phenolic composition, antioxidant activity, storage stability, and in vitro gastrointestinal bioaccessibility using the INFOGEST 2.0 protocol. Among the extraction conditions investigated, HPAE for 30 min (HPAE30) produced the highest total phenolic content (TPC), reaching 133.71 ± 1.14 mg GAE/g dry matter, compared with 31.68 ± 0.16 mg GAE/g for UAE30 and 63.78 ± 0.83 mg GAE/g for sequential UAE15–HPAE30. HPAE30 also showed the highest DPPH antioxidant activity (82.52 ± 0.01 μM TE/g dry matter), whereas the sequential treatment showed lower DPPH activity (32.70 ± 0.02 μM TE/g). HPAE30 was therefore selected for subsequent spray drying. The resulting powder showed an encapsulation efficiency of 34.26%, while EP exhibited lower water activity (0.35 ± 0.002) than NEP (0.65 ± 0.001). During 14 days of storage under light exposure, TPC decreased from 39.01 ± 0.33 to 30.15 ± 0.50 mg GAE/g in NEP, whereas EP retained approximately 95% of its initial TPC. Following in vitro gastrointestinal digestion, the TPC‐based intestinal bioaccessibility index was 66% for EP compared with 58% for NEP. Overall, HPAE30 provided the highest recovery of phenolic compounds under the conditions investigated, while alginate–xanthan encapsulation improved the retention of phenolic compounds during storage and was associated with higher in vitro intestinal bioaccessibility compared with the non‐encapsulated powder.

## Graphical abstract

Alginate–xanthan encapsulation of HPAE30 date seed extract improved the retention of phenolic compounds during storage and enhanced their in vitro intestinal bioaccessibility.

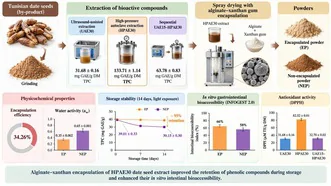

## 1 Introduction

The transition toward more sustainable food systems has strengthened interest in circular‐economy approaches that convert agro‐industrial by‐products into value‐added ingredients while reducing resource losses and environmental burdens. In this context, plant‐derived residues are increasingly recognized as promising sources of bioactive compounds that can be recovered through more sustainable processing technologies. Recent literature has particularly emphasized the recovery of polyphenol‐rich extracts from agro‐industrial by‐products as a strategy consistent with both green chemistry and circular bioeconomy principles (Bernini et al. 2024).

Date seeds ( *Phoenix dactylifera* L.) are an abundant but comparatively underutilized by‐product of the date‐processing sector. Their valorization is of particular interest because date seeds contain phenolic acids, flavonoids, condensed tannins, and other phytochemicals associated with antioxidant activity. Recent studies have demonstrated the potential of date seeds as a source of bioactive compounds using alternative extraction technologies. For example, ultrasound‐assisted extraction using natural deep eutectic solvents has been reported to enhance the recovery of phenolic compounds and antioxidant activity from date seeds, while comparative studies have demonstrated the potential of ultrasound‐ and microwave‐assisted extraction as alternatives to conventional extraction approaches (Airouyuwa et al. 2022). More recently, aqueous ultrasound‐assisted extraction of defatted date seeds was reported as an effective approach for recovering bioactive compounds, further supporting the potential of ultrasound‐based processes for date‐seed valorization (Al‐Farsi and Lee 2008). More recent work has further corroborated ultrasound‐assisted extraction as an efficient route for recovering phenolic compounds from date seed matrices, reporting extraction yields and antioxidant capacities comparable to conventional solvent extraction (Shi et al. 2023), and has systematically identified extraction amplitude, time, and liquid‐to‐solid ratio as key parameters governing (poly)phenol recovery from date palm seeds (Lucas‐González et al. 2024).

Polyphenols constitute an important group of plant secondary metabolites and have attracted considerable attention because of their antioxidant and other biological activities. However, their recovery and subsequent application in food and nutraceutical systems depend strongly on the extraction technology and processing conditions employed. Conventional solid–liquid extraction methods, including prolonged maceration and Soxhlet extraction, may involve high solvent consumption, extended processing times, and substantial thermal exposure. Consequently, alternative extraction technologies have been increasingly investigated to improve mass transfer and compound recovery while reducing solvent and energy requirements (Bernini et al. 2024; Chemat et al. 2019). Ultrasound‐assisted extraction (UAE) is one of the most widely investigated green extraction technologies. Acoustic cavitation generated during sonication can enhance solvent penetration and mass transfer by disrupting plant tissues and facilitating the release of intracellular compounds. Its application to date seeds has already demonstrated its potential for improving phenolic recovery under aqueous or alternative solvent systems (Airouyuwa et al. 2022). High‐pressure or hydrothermal extraction approaches provide a complementary mechanism by exposing plant matrices to elevated pressure and temperature, which can facilitate solvent penetration and matrix disruption. However, the relative effectiveness of these approaches depends on the nature of the plant matrix and on the applied processing conditions. Therefore, comparative evaluation of different extraction strategies under controlled conditions remains necessary for identifying an appropriate process for a specific agro‐industrial residue.

An additional challenge is that the recovery of polyphenols does not necessarily guarantee their stability during subsequent processing and storage. Phenolic compounds may undergo degradation or transformation when exposed to oxygen, light, elevated temperature, and other environmental factors. Consequently, the development of a suitable stabilization strategy is an important step toward their incorporation into functional food or nutraceutical formulations. Spray‐drying is particularly attractive because it is widely applicable to food ingredients and can convert liquid extracts into more stable powders through incorporation into a protective carrier matrix. Encapsulation can potentially reduce the exposure of sensitive compounds to environmental stressors and modify their release during gastrointestinal digestion. Nevertheless, extraction and stabilization are often investigated as separate processing stages. A more integrated approach is therefore needed to determine whether optimization of the extraction step, followed by encapsulation of the resulting extract, can improve the stability and gastrointestinal accessibility of date‐seed phenolics. In particular, recent research on date‐seed extraction has mainly focused on optimizing individual extraction technologies or solvent systems (Airouyuwa et al. 2022), whereas comparatively limited attention has been given to linking extraction‐process selection with subsequent spray‐drying stabilization and in vitro gastrointestinal assessment.

The present study was therefore designed to evaluate three extraction strategies for Tunisian date seeds: ultrasound‐assisted extraction (UAE), high‐pressure autoclave extraction (HPAE), and a sequential UAE followed by HPAE treatment. The extraction treatments were compared in terms of total phenolic content, total flavonoid content, total condensed tannins, and antioxidant activity. The most effective extraction treatment was subsequently selected for spray‐drying using an alginate–xanthan gum carrier system. The resulting encapsulated and non‐encapsulated powders were characterized and evaluated under different light and temperature storage conditions. Finally, their phenolic release and antioxidant properties were assessed following simulated gastrointestinal digestion using the INFOGEST 2.0 protocol, a standardized in vitro digestion approach designed to improve the physiological relevance and comparability of gastrointestinal digestion studies (Brodkorb et al. 2019; Silva et al. 2024). Accordingly, the study addresses an integrated research gap at the interface of green extraction, date‐seed by‐product valorization, spray‐drying stabilization, and gastrointestinal bioaccessibility, while providing experimental evidence on the performance of different extraction strategies and the potential contribution of encapsulation to the stability and in vitro accessibility of date‐seed phenolics.

## 2 Materials and Methods

### 2.1 Plant Material and Preparation

Date palm seed powder ( *Phoenix dactylifera* L.) was provided by VAPACA Boudjbal a date‐processing company located in Tunisia. The powder was obtained from date seeds generated as a by‐product of date processing. Upon receipt, the powder was stored in hermetically sealed dark polyethylene bags at −20°C until further analysis.

### 2.2 Chemicals and Reagents

Folin–Ciocalteu reagent, gallic acid (≥ 98%), quercetin (≥ 98%), DPPH• (2,2‐diphenyl‐1‐picrylhydrazyl), ABTS•^+^ (2,2′‐azino‐bis(3‐ethylbenzothiazoline‐6‐sulphonic acid)), potassium persulphate, Trolox (±6‐hydroxy‐2,5,7,8‐tetramethylchromane‐2‐carboxylic acid), AlCl₃, sodium carbonate, ethanol (HPLC grade), methanol (HPLC grade), and maltodextrin (DE 14–20) were purchased from Sigma‐Aldrich (St. Louis, MO, USA). All enzymes used for the INFOGEST protocol (porcine pepsin ≥ 2500 U/mg, pancreatin 8× USP, bovine bile extract) were obtained from Sigma‐Aldrich. Ultra‐pure water (18.2 MΩ cm) produced by a Milli‐Q system (Millipore, USA) was used throughout.

### 2.3 Green Extraction Methods

#### 2.3.1 Ultrasound‐Assisted Extraction (UAE)

Ultrasound‐assisted extractions were performed using a Bransonic ultrasonic cleaner model 3210 (Branson, USA), with a resonance frequency of 47 kHz and an acoustic power output of 120 W. Each sample consisted of 1 g of date seed powder homogeneously suspended in 10 mL of distilled water. The temperature was monitored throughout sonication and maintained at 30°C to minimize thermal degradation of heat‐sensitive phenolic compounds.

#### 2.3.2 High‐Pressure Autoclave Extraction (HPAE)

High‐pressure autoclave extraction (HPAE) was performed using a stainless‐steel pressure‐cooker autoclave (6 L capacity; maximum operating pressure, 1.5 bar; maximum temperature, 120°C). Briefly, 1 g of date seed powder was suspended in 10 mL of distilled water and placed in a sealed glass vessel inside the autoclave. The system was gradually pressurized to an operating pressure of 1.0 bar gauge pressure (approximately 2.0 bar absolute). Under these operating conditions, the extraction temperature was maintained at approximately 120°C. Extraction was conducted for 15 or 30 min, depending on the treatment. The pressure was controlled through the autoclave pressure‐regulation system and maintained throughout the designated extraction period. The extraction procedure was adapted from the pressure‐assisted polyphenol extraction approach described by Zhang et al. (2019). Immediately after completion of the extraction period, the samples were rapidly removed from the autoclave and quench‐cooled in an ice bath to minimize further thermal exposure and potential degradation of thermolabile bioactive compounds.

#### 2.3.3 Combined UAE/HPAE

The combined UAE‐HPAE method consisted of the sequential application of ultrasonication (§2.3.1) followed immediately by high‐pressure autoclave extraction (§2.3.2), without intermediate filtering. The extraction duration for each step was set according to the optimal conditions determined from preliminary optimisation experiments. This sequential approach was specifically designed to exploit the synergistic interplay between acoustic cavitation‐mediated cell wall disruption during the UAE step and the subsequent pressure‐enhanced solvent penetration during HPAE, thereby maximizing the release and recovery of bioactive compounds from date seed powder, a rationale conceptualized for hybrid green extraction systems (Nguyen and Karboune 2023).

#### 2.3.4 Filtration and Recovery

After each extraction, suspensions were filtered through Whatman No. 1 filter paper and then centrifuged using a refrigerated centrifuge (for 4000 × *g*, 10 min, at 4°C). The supernatants (crude extracts) were collected, adjusted to a standard volume, aliquoted, and stored at −20°C. All extractions were performed in triplicate on three independent batches. Three independent extraction batches were prepared, and each batch was analyzed in triplicate. The three independently prepared batches were considered the experimental replicates, whereas the triplicate measurements were considered analytical replicates.

### 2.4 Determination of Total Polyphenol Content (TPC)

Total phenolic content (TPC) was determined using the Folin–Ciocalteu method originally described by Singleton and Rossi (1965) and colorimetric method as described by Rebai et al. (2025), with minor modifications. An aliquot of diluted extract (100 μL) was reacted with Folin–Ciocalteu reagent (10%) and Na_2_CO_3_ solution (500 μL, 7.5% w/v), then incubated for 30 min at room temperature in the dark. Absorbance was measured at 765 nm with UV/VIS spectrophotometer (MecasysOptizen Pop, Mecasys Co. Ltd., Daejeon, Korea). Results were quantified against a gallic acid calibration curve (0–200 μg/mL; *R* ^2^ = 0.999) and expressed as mg gallic acid equivalents per gram dry matter.

### 2.5 Determination of Total Flavonoid Content (TFC)

Total flavonoid content (TFC) was determined by the AlCl₃ originally developed by Zhishen et al. (1999). Absorbance was recorded at 510 nm. Results were quantified using a quercetin calibration curve (0–500 μg/mL; *R* ^2^ > 0.998) and expressed as mg quercetin equivalents per gram dry matter (mg QE/g DM).

### 2.6 Determination of Total Condensed Tannins Content (TCT)

The total condensed tannin (TCT) content was determined using the vanillin‐HCl method, originally developed by Broadhurst and Jones (1978). Briefly, 250 μL of extract was mixed with 1500 μL of a 4% vanillin solution (w/v), followed by the addition of 750 μL of concentrated hydrochloric acid (HCl). The mixture was incubated at room temperature for 15 min, and the absorbance was subsequently measured at 500 nm using a spectrophotometer.

### 2.7 Measurement of Total Antioxidants Activity

The free radical scavenging capacity was evaluated using the stable 1,1‐diphenyl‐2‐picrylhydrazyl (DPPH) radical assay (Brand‐Williams et al. 1995). An aliquot of 0.50 mL of extract was combined with 0.95 mL of DPPH methanolic solution and incubated in the dark at room temperature for 30 min. Absorbance was subsequently measured at 517 nm, using methanol as blank. The radical scavenging activity was calculated and results expressed as Trolox equivalents per gram dry matter (μmol TE/g DM).

The ABTS^+^ radical cation decolorization assay was performed according Re et al. (1999). Absorbance was recorded at 734 nm against an appropriate blank, and results were expressed as mg Trolox equivalents per gram dry matter (μmol TE/g DM).

### 2.8 Microencapsulation by Spray‐Drying

#### 2.8.1 Feed Solution Preparation

The optimal date seed extract, selected based on its total phenolic content and antioxidant activity, was combined with alginate and xanthan gum at an alginate‐to‐xanthan ratio of 1:3 (w/w) (Iranloye et al. 2026). The mixture was magnetically stirred at room temperature for 30 min to promote homogenization of the feed solution. The resulting mixture was then passed through a 100 μm membrane filter to remove any undissolved particles prior to spray drying. The spray‐drying process was performed using the prepared feed solution. The resulting powder was subsequently subjected to physicochemical and phytochemical analyses, with each analytical measurement performed in triplicate. The triplicate measurements were considered analytical replicates and were not treated as independent experimental observations.

#### 2.8.2 Spray‐Drying Conditions

Spray‐drying was carried out using a laboratory‐scale spray dryer (GIPP‐2000, Shanghai GIPP, China), equipped with a two‐fluid nozzle atomization system and a built‐in oil‐free air compressor. The drying chamber, cyclone separator, and collection system were made of high‐quality borosilicate glass to ensure a contamination‐free process and easy cleaning. The operating conditions were set as follows: inlet air temperature of 160°C and outlet temperature ranging between 70°C and 80°C. The feed solution was delivered using a peristaltic pump at a flow rate of 5 mL/min, with an aspirator rate of 100%. Atomization was achieved using compressed air at a flow rate of 473 L/h through a stainless‐steel nozzle with a diameter of 0.7 mm. The system operates with real‐time PID temperature control (±1°C accuracy), ensuring stable thermal conditions throughout the process. The average drying time was estimated between 0.8 and 1.5 s, minimizing thermal degradation of heat‐sensitive bioactive compounds. The maximum evaporation capacity of the system ranged from 1500 to 2000 mL/h, depending on the feed composition. The spray dryer was operated in automatic mode, allowing precise control of parameters via a digital interface. A cyclone separator and an additional bag collection system were used to improve powder recovery efficiency. The resulting powders exhibited a relatively uniform particle size distribution, with more than 95% of particles within a consistent size range. These conditions were selected based on previous studies reporting optimal spray‐drying parameters for polyphenol‐rich and heat‐sensitive compounds (Hashim et al. 2022).

### 2.9 Characterization of Spray‐Dried Powders

The physicochemical characterization of the spray‐dried powder was carried out through a comprehensive set of analytical determinations.

Moisture content was determined gravimetrically by oven drying at 105°C to constant weight, in accordance with the protocols of Farahnaky et al. (2016). Water activity (a_w_) was measured at 25°C using an AquaLab Series 4TE water activity meter (Decagon Devices, USA).

Water solubility index (WSI) was determined according to Jafari et al. (2017) with minor modifications. Briefly, 2.5 g of powder was suspended in 30 mL of distilled water in a tared centrifuge tube and homogenized using a vortex mixer for 1 min. The suspension was then incubated in a water bath at 37°C for 30 min, followed by centrifugation at 3500 rpm and 4°C for 20 min. The resulting supernatant was carefully decanted into a pre‐weighed dish and dried at 105°C to constant weight. WSI was calculated as follows:

$$\mathrm{WSI}\;\left(\%\right)=\left(\text{Dried supernatant weight}/\text{Initial sample weight}\right)\times 100$$

Color parameters (L*, a*, b*) were measured using a calibrated colorimeter (Shenzhen 3nh Technology Co. Ltd.). Soluble solids content (°Brix) was determined at ambient temperature using a portable digital refractometer (PAL, ATAGO, Japan).

Encapsulation efficiency (EF): The encapsulation efficiency (EF) corresponds to the fraction of total polyphenols effectively entrapped within the core of the wall material. It was determined according to the protocol described by Ranasinghe et al. (2025), based on the total phenolic content (TPC) of the defatted date seed powder extract (TPC_ext) and the surface phenolic content of the encapsulated powder (TPC_surface).

$$\mathrm{EF}\;\left(\%\right)=\left({\mathrm{TPC}}_{\mathrm{ext}}–{\mathrm{TPC}}_{\text{surface}}\right)/{{\mathrm{TPC}}_{\mathrm{ext}}}^{*}\;100$$

Antioxidant activity: The determination of total bioactive compounds (phenolics, flavonoids, and tannins) and radical scavenging capacities (DPPH and ABTS) was conducted on the obtained powders as detailed above. All measurements were performed in triplicate.

### 2.10 Storage Stability: Effects of Light Exposure and Temperature

To evaluate storage stability, both encapsulated (EP) and non‐encapsulated (NEP) powders were stored under different light and temperature conditions for 14 days. Samples were maintained at 25°C and 40°C under two light conditions: continuous light exposure and total darkness. Samples were collected at Day 0, Day 7, and Day 14 to evaluate changes in total phenolic content (TPC) and antioxidant activity, as determined by the DPPH and ABTS assays. The storage conditions were established to compare the stability of EP and NEP powders under the investigated combinations of temperature and light exposure. The study therefore focused on the observed changes in bioactive‐compound retention and antioxidant activity during storage rather than on assigning specific degradation mechanisms. For each storage condition, three independently prepared samples/batches were evaluated at Days 0, 7, and 14. Each sample was analyzed in triplicate. The independently prepared samples/batches were considered the experimental replicates, whereas the repeated analytical measurements were considered technical/analytical replicates and were not treated as independent observations. The storage period was consistently defined as 14 days, with Day 0 representing the initial measurement before storage.

### 2.11 In Vitro Gastrointestinal Digestion‐ INFOGEST 2.0 Protocol

The in vitro bioaccessibility of phenolic compounds was assessed following the international harmonized INFOGEST 2.0 static protocol (Brodkorb et al. 2019). This method was selected for its high correlation with in vivo human digestion data concerning polyphenol‐rich matrices (Morales et al. 2025). All enzyme activities were verified spectrophotometrically according to the specific assays described in the INFOGEST recommendations prior to the experiment.

The process involved three sequential stages:

Oral phase: The encapsulated and non‐encapsulated powders were mixed 1:1 (v/v) with SSF containing porcine salivary alpha‐amylase (75 U/mL). The mixture was incubated for 2 min at pH 7.0.

Gastric phase: The resulting oral bolus was diluted 1:1 (v/v) with simulated gastric fluid (SGF). The final mixture contained porcine pepsin (2000 U/mL), CaCl_2_ (0.075 mM), and the pH was adjusted to 3.0 using 1 M HCl and incubated for 2 h at 37°C.

Intestinal phase: The gastric chyme was neutralized to pH 7.0 with NaOHand mixed 1:1 (v/v) with simulated intestinal fluid (SIF). The final intestinal content reached concentrations of pancreatin (100 U/mL based on trypsin activity), bile salts (10 mM), and CaCl_2_ (0.6 mM). Digestion proceeded for 2 h and was terminated by heating at 95°C for 5 min to irreversibly inactivate the enzymes, followed by immediate cooling on ice.

Post‐digestion, aliquots from each phase were centrifuged (3000*g, 10 min, 4°C) and the supernatants were filtered through a 0.45 μm membrane. The phenolic compounds and antioxidant activity were quantified in the supernatants.

The bioaccessibility index (BI) was calculated using the following equation:

$$\mathrm{BI}\;\left(\%\right)=\left[\left({\mathrm{TPC}}^{\text{intestinal}}\right)/\left({\mathrm{TPC}}^{\text{initial}}\right)\right]\times 100$$

The INFOGEST digestion procedure was performed in three independent digestion runs. Samples from each digestion stage were analyzed in triplicate. The independent digestion runs were considered the experimental replicates, whereas the repeated measurements were considered analytical replicates. Throughout the study, analytical triplicates were not considered independent observations; statistical analyses were based on the appropriate experimental unit for each experiment.

### 2.12 Statistical Analysis

All experimental measurements were carried out in triplicate and results are expressed as mean ± standard deviation. All analyses were performed with IBM SPSS Statistics v.26 (IBM Corp., USA) and GraphPad Prism v.9.5. Data from three independent experiments were subjected to one‐way analysis of variance (ANOVA) followed by Tukey's test, with a significance threshold of *p* < 0.05.

## 3 Results and Discussion

### 3.1 Effect of Green Extraction Methods on Total Polyphenol Content, Flavonoid Content, and Antioxidant Activity of Date Seed Extracts

The phytochemical composition and antioxidant potential of date pit extracts obtained by ultrasound‐assisted extraction (UAE), high‐pressure autoclave extraction (HPAE), and their combination (UAE15′‐HPAE30′) were evaluated and are presented in Table 1. HPAE30′ yielded the highest value for total polyphenol content TPC (133.71 ± 1.14 mg GAE/g), significantly exceeding both UAE30′ (31.68 ± 0.16 mg GAE/g) and the combined UAE15′‐HPAE30′ treatment (63.78 ± 0.83 mg GAE/g). Notably, the combined extraction did not surpass HPAE alone, suggesting that the lower phenolic recovery observed under some ultrasound‐assisted conditions may reflect differences in extraction efficiency and compound stability under the applied processing conditions. Oxidative degradation may represent one possible contributing mechanism; however, it was not directly investigated in the present study. A consistent trend was observed for total flavonoid content (TFC) and total condensed tannins (TCT), where HPAE30′ again showed the highest value, while UAE30′ recorded the lowest and the combined treatment yielded an intermediate value (*p* < 0.05). Contrary to the hypothesis proposed Chemat et al. (2019), which suggests that UAE pre‐treatment enhances solvent accessibility during subsequent high‐pressure extraction through acoustic cavitation‐induced cell wall disintegration, our results did not support this synergistic effect.

**TABLE 1:** Total phenolic content (TPC), total flavonoid content (TFC), total condensed tannin content (TCT), and antioxidant activity of date‐seed extracts obtained using different extraction treatments.

Parameter	UAE 15 min	UAE 30 min	HPAE 15 min	HPAE 30 min	Sequential UAE15–HPAE30
TPC (mg GAE/g DM)	31.22 ± 0.018	31.68 ± 0.16	97.65 ± 0.41	133.71 ± 1.14	63.78 ± 0.83
TFC (mg QE/g DM)	1.29 ± 0.05	0.61 ± 0.03	30.40 ± 0.26	44.96 ± 0.18	12.44 ± 0.33
TCT (mg CE/g DM)	355.40 ± 2.04	359.40 ± 1.46	368.06 ± 1.24	551.40 ± 3.86	360.73 ± 1.04
DPPH (μM Trolox/g DM)	62.55 ± 0.02	53.49 ± 0.03	73.28 ± 0.007	82.52 ± 0.01	32.70 ± 0.02
ABTS (μM Trolox/g DM)	20.80 ± 0.012	32.85 ± 0.012	30.48 ± 0.044	61.50 ± 0.009	60.94 ± 0.004

Abbreviations: CE, catechine quivalents; DM, dry matter; GAE, gallicacidequivalents; HPAE, high‐pressure autoclave extraction; QE, quercetin equivalents; TE, Trolox equivalents; UAE, ultrasound‐assisted extraction.

Regarding antioxidant activity assessed by the DPPH radical scavenging assay, HPAE30′ exhibited the strongest capacity (82.52 ± 0.01 μM TE/g), whereas the combined UAE15′‐HPAE30′ showed the lowest value (32.70 ± 0.02 μM TE/g), with UAE30′ presenting an intermediate activity (53.49 ± 0.03 μM TE/g) (*p* < 0.05). In contrast, ABTS‐based antioxidant capacity revealed a markedly different pattern, where the combined UAE15′‐HPAE30′ (60.94 ± 0.004 μM Trolox/g) and HPAE30′ (61.5 ± 0.009 μM TE/g) showed comparable and highest values, while UAE15′ recorded the lowest ABTS activity (*p* < 0.05), improving method‐dependent differences in radical scavenging mechanisms. These divergent DPPH and ABTS results suggest that the extracts contain both hydrophilic and lipophilic antioxidant compounds that respond differently depending on the extraction technique applied. To further investigate the relationship between phenolic composition and antioxidant potential, Pearson correlation coefficients were calculated between TPC, TFC, and the two antioxidant assays (DPPH and ABTS). All correlations were positive and moderately strong, with *r* values ranging from 0.718 to 0.772 (*p* < 0.05), suggesting that phenolic compounds, including flavonoids, are major contributors to the antioxidant capacity of date pit extracts. Specifically, TPC showed a significant positive correlation with both DPPH (*r* = 0.738) and ABTS (*r* = 0.753) radical scavenging activities, indicating that extracts richer in total polyphenols tend to exhibit stronger free radical neutralization capacity, regardless of the assay used. Similarly, TFC was positively correlated with DPPH (*r* = 0.772) and ABTS (*r* = 0.718) activities, with the slightly higher *r* value observed for the DPPH assay suggesting that flavonoids may contribute more directly to hydrogen atom transfer mechanisms than to electron transfer‐based scavenging. These findings are consistent with previous studies reporting strong correlations between polyphenol content and antioxidant activity in date pit extracts (Khalid et al. 2021), and confirm that the observed differences in antioxidant capacity across extraction methods are largely attributable to variations in phenolic compound recovery. Nevertheless, the moderate rather than strong correlation values (*r* < 0.80) suggest that other bioactive compounds, such as carotenoids, tocopherols, or dietary fiber‐bound antioxidants, may also contribute to the overall antioxidant potential of date pit extracts, as previously reported by Mrabet et al. (2022). Overall, HPAE30 consistently produced the highest TPC, TFC, and TCT values and the highest DPPH activity, while also providing the highest ABTS activity. These results indicate that the high‐pressure treatment was the most effective of the investigated extraction conditions for recovering the measured phytochemical compounds and antioxidant activity from date seeds. The superior performance of HPAE30 may be associated with enhanced disruption of the date seed matrix and improved release of intracellular or matrix‐associated compounds. However, the specific physicochemical mechanisms responsible for this enhanced extraction were not directly investigated in the present study. Based on its overall extraction performance, HPAE30 was therefore selected for the subsequent encapsulation experiments.

### 3.2 Physicochemical Characterization of Encapsulated and Non‐Encapsulated Date Pit Powders

The physicochemical properties of the encapsulated (EP) and non‐encapsulated (NEP) date pit powders were evaluated in terms of powder yield, water solubility index (WSI), water activity (aw) moisture content, and color parameters (L*, a*, and b*). The results are presented in Table 2. The powder yield was 6.15% for EP compared with 2.50% for NEP, indicating a higher powder recovery following spray drying in the presence of the alginate–xanthan gum wall matrix. The WSI was significantly higher for NEP (9.560% ± 0.003%) than for EP (3.088% ± 0.001%) (*p* < 0.05). The lower WSI observed for EP may be related to the presence of the wall‐material matrix, which can modify the interaction of the encapsulated extract with water and consequently affect powder reconstitution and solubilization. However, the specific contribution of matrix composition to the observed difference in solubility was not directly investigated in the present study. Water activity (aw) was markedly lower in EP (0.35 ± 0.002) than in NEP (0.65 ± 0.001), indicating lower availability of unbound water in the encapsulated powder. Lower water activity is generally considered an important quality parameter for improving the storage stability of dried food ingredients because it can influence the rates of microbial growth and chemical reactions. The lower aw observed for EP may therefore contribute to its greater stability during storage (Krishnan et al. 2005). However, water activity alone does not establish the occurrence or rate of specific degradation reactions. Moisture content was also lower for EP (0.428% ± 0.001%) than for NEP (1.713% ± 0.001%). This difference indicates distinct moisture‐related behavior between the two powder formulations and may reflect differences in the interaction of the powders with environmental moisture. Nevertheless, the present measurements do not allow the specific physicochemical mechanism responsible for this difference to be established.

**TABLE 2:** Physicochemical characterization of encapsulated (EP) and non‐encapsulated (NEP) spray‐dried powders (mean ± SD, *n* = 3).

	EP	NEP
Yield %	6.15	2.5
Water soluble Index (%WSI)	3.088 ± 0.001	9.560 ± 0.003
Water activity (Aw)	0.35 ± 0.002	0.65 ± 0.001
Moisture content	0.428 ± 0.001	1.713 ± 0.001
L*	62.75 ± 0.01	67.09 ± 0.03
a*	13.07 ± 0.01	13.59 ± 0.01
b*	17.20 ± 0.02	20.42 ± 0.03
EE %	34.26%	—

Color measurements showed significant differences between EP and NEP (*p* < 0.05). The L* value was lower for EP (62.75 ± 0.01) than for NEP (67.09 ± 0.03), indicating a darker appearance of the encapsulated powder. The a* values were positive for both formulations, with slightly lower redness for EP (13.07 ± 0.01) than for NEP (13.59 ± 0.01). Similarly, the b* value was lower for EP (17.20 ± 0.02) than for NEP (20.42 ± 0.03), indicating a difference in the yellow component of the powder color. The observed color differences may be related to differences in the relative contribution of the date pit extract and the alginate–xanthan gum wall matrix to the overall surface color of the powders. Changes in color may also be influenced by the thermal conditions applied during spray drying. Although Maillard‐type reactions could potentially contribute to color development during thermal processing, this mechanism was not directly investigated in the present study; therefore, the observed color differences cannot be attributed specifically to Maillard reactions. Similarly, the lower b* value of EP should not be interpreted as direct evidence of masking of specific pigments, since individual pigment or phenolic compounds were not quantitatively characterized in the present study.

Thus, encapsulation resulted in clear differences in the physicochemical properties of the date pit powders, particularly in water activity, water solubility, and color parameters. The lower aw observed for EP suggests a potentially more favorable physicochemical state for storage compared with NEP. These findings are consistent with a protective contribution of the alginate–xanthan gum matrix; however, the specific physicochemical mechanisms underlying the observed differences were not directly characterized. Further studies including detailed moisture sorption analysis, morphological characterization, and evaluation of matrix‐related properties would be useful to better understand the behavior and stability of the encapsulated powder.

The encapsulation efficiency obtained in the present study was 34.26%, which was relatively low compared with values reported for several spray‐dried phenolic‐rich systems. Previous studies have reported encapsulation efficiencies of approximately 81.5% for phenolic acids and derivatives encapsulated using hydroxypropyl‐β‐cyclodextrin and maltodextrin by spray drying, with substantial variability among individual phenolic compounds (Escobar‐Avello et al. 2021). Similarly, encapsulation efficiencies as high as 98.83% have been reported for spray‐dried ciriguela peel phenolic extracts using maltodextrin and gum arabic as encapsulating agents (Da Silva Júnior et al. 2023). More recently, grape pomace phenolic extracts encapsulated using alginate‐based coatings achieved EE values of 95.90%–98.01% when sodium alginate was combined with gelatin, whereas lower efficiencies were obtained when sodium alginate was used alone (Martinović et al. 2025). These differences highlight the strong influence of wall‐material composition and spray‐drying formulation on phenolic retention. The comparatively lower EE observed in the present study may be associated with differences in wall‐material composition, core‐to‐wall ratio, feed solids concentration, and spray‐drying conditions. The alginate–xanthan gum matrix may also interact differently with phenolic compounds than maltodextrin‐ or gum‐arabic‐based systems. However, these factors were not systematically investigated in the present study and therefore cannot be identified as direct causes of the observed EE. Further optimization of the formulation and spray‐drying parameters is warranted to improve encapsulation performance.

### 3.3 Phytochemical Characterization and Antioxidant Activity of Encapsulated and Non‐Encapsulated Date Seed Powders

The phytochemical characteristics and antioxidant activity of encapsulated (EP) and non‐encapsulated (NEP) date seed powders are presented in Table 3. The encapsulated powder exhibited a significantly higher total phenolic content (TPC) of 45.816 ± 0.17 mg GAE/g compared with 39.013 ± 0.33 mg GAE/g for the non‐encapsulated powder, corresponding to an increase of approximately 17.4%. This increase may indicate that the encapsulation process contributed to the preservation and/or improved extractability of phenolic compounds during processing and subsequent analysis. This finding is in agreement with those reported by Chan et al. (2024), who demonstrated that microencapsulation of oil palm leaf extract by spray drying using maltodextrin retained up to 81.52% of total phenolic content, confirming the protective role of the encapsulant matrix against phenolic degradation.

**TABLE 3:** Phytochemical characterization and antioxidant activity of encapsulated and non‐encapsulated date seed powders.

	TPC (mg GAE/g DM)	TFC (mg QE/g DM)	TCT (mg CE/g DM)	DPPH activity (μM TE/g DM)	ABTS activity (μM TE/g DM)
EP	45.816 ± 0.17	1.76 ± 0.004	360.85 ± 0.21	84.049 ± 0.014	103.81 ± 0.002
NEP	39.013 ± 0.33	0.745 ± 0.001	256.683 ± 0.44	83.016 ± 0.017	100.835 ± 0.009

Abbreviations: CE, catechin equivalents; DM, dry matter; GAE, gallic acid equivalents; QE, quercetin equivalents; TE, Trolox equivalents.

Similarly, the total flavonoid content (TFC) was markedly higher in EP (1.76 ± 0.004 mg QE/g) than in NEP (0.745 ± 0.001 mg QE/g). The approximately 136% increase observed after encapsulation suggests that the encapsulating matrix may have contributed to the stabilization of flavonoid compounds and/or enhanced their recovery during the analytical procedure. Such effects may depend strongly on the chemical nature of the wall material and its interactions with the encapsulated phytochemicals. The total condensed tannin content (TCT) followed the same trend, with EP showing a markedly higher value (360.85 ± 0.21 mg CE/g) than NEP (256.683 ± 0.44 mg CE/g). The higher TCT value in the encapsulated powder may similarly reflect improved protection of tannin‐related compounds during processing and enhanced recovery from the encapsulated matrix. However, because the present study did not separately characterize individual tannin compounds or investigate their stability throughout the encapsulation process, this interpretation should be considered cautiously. Regarding antioxidant activity, EP demonstrated slightly higher radical‐scavenging activity against both DPPH (84.049 ± 0.014 μM TE/g) and ABTS (103.81 ± 0.002 μM TE/g) compared with NEP (83.016 ± 0.017 and 100.835 ± 0.009 μM TE/g, respectively). The higher antioxidant activity observed for EP is consistent with its higher TPC, TFC, and TCT values, as phenolic compounds and flavonoids can contribute substantially to radical‐scavenging capacity. Previous studies have also demonstrated that encapsulation can preserve the antioxidant activity of phenolic‐rich extracts during drying. For instance, spray‐dried ciriguela peel extracts exhibited high antioxidant preservation when appropriate wall materials were employed. Similarly, alginate‐based encapsulation systems have been reported to provide high encapsulation efficiencies for grape pomace phenolic extracts, although performance varied according to the composition of the coating material (Martinović et al. 2025). Overall, the present results indicate that encapsulation was associated with higher measured levels of phenolic compounds, flavonoids, and condensed tannins, together with a modest improvement in DPPH and ABTS radical‐scavenging activities. These findings suggest that the encapsulation system used in the present study may contribute to the stabilization and improved recovery of bioactive compounds from date seed powder. Nevertheless, the observed increases should not be interpreted exclusively as evidence of chemical preservation, since changes in extractability and analytical accessibility following encapsulation may also contribute to the differences between EP and NEP. Further studies involving storage stability, individual phenolic profiling, and controlled comparisons before and after encapsulation would be required to confirm the protective effect of the encapsulation matrix.

### 3.4 Storage Stability of Bioactive Compounds and Antioxidant Potential of Encapsulated and Non‐Encapsulated Date Seed Powders

The storage stability of encapsulated (EP) and non‐encapsulated (NEP) date seed powders was evaluated over a 14‐day period under different environmental conditions, including light exposure, darkness, and storage temperatures of 25°C and 40°C. The stability of the powders was assessed by monitoring total phenolic content (TPC), total flavonoid content (TFC), total condensed tannin content (TCT), and antioxidant activity determined by DPPH and ABTS assays. The results are presented as mean ± standard deviation (SD), and changes during storage were evaluated descriptively based on the variation in mean values and percentage retention relative to the corresponding Day 0 values.

#### 3.4.1 Effect of Light Exposure on Storage Stability

Light exposure was associated with a more pronounced decrease in the measured bioactive compounds in NEP than in EP. After 14 days of storage under light, TPC in NEP decreased from 39.01 ± 0.33 mg GAE/g at Day 0 to 30.15 ± 0.50 mg GAE/g, corresponding to a retention of approximately 77.3% of the initial value. In comparison, EP retained 43.50 ± 0.25 mg GAE/g at Day 14, corresponding to approximately 95.0% retention of its initial TPC (Figure 1). A similar pattern was observed for TFC and TCT. At Day 14 under light exposure, EP retained approximately 93.8% and 92.9% of its initial TFC and TCT, respectively, whereas NEP retained approximately 64.9% and 74.2%. The antioxidant assays showed a comparable trend, with EP retaining approximately 96.6% of its initial DPPH activity and 94.9% of its ABTS activity, compared with 84.8% and 81.6%, respectively, for NEP. Overall, the descriptive retention profiles indicate that the encapsulated powder was less affected by light exposure than the non‐encapsulated powder. The comparatively higher retention observed for EP may be related to the physical protection provided by the alginate–xanthan gum matrix, which may reduce the direct exposure of the encapsulated compounds to light and oxygen. The wall material may therefore contribute to a shading or barrier effect that limits photo‐induced degradation of susceptible phenolic constituents. However, because the physicochemical mechanisms underlying this protection were not directly characterized in the present study, this interpretation should be considered a possible explanation rather than an experimentally demonstrated mechanism.

**FIGURE 1:**
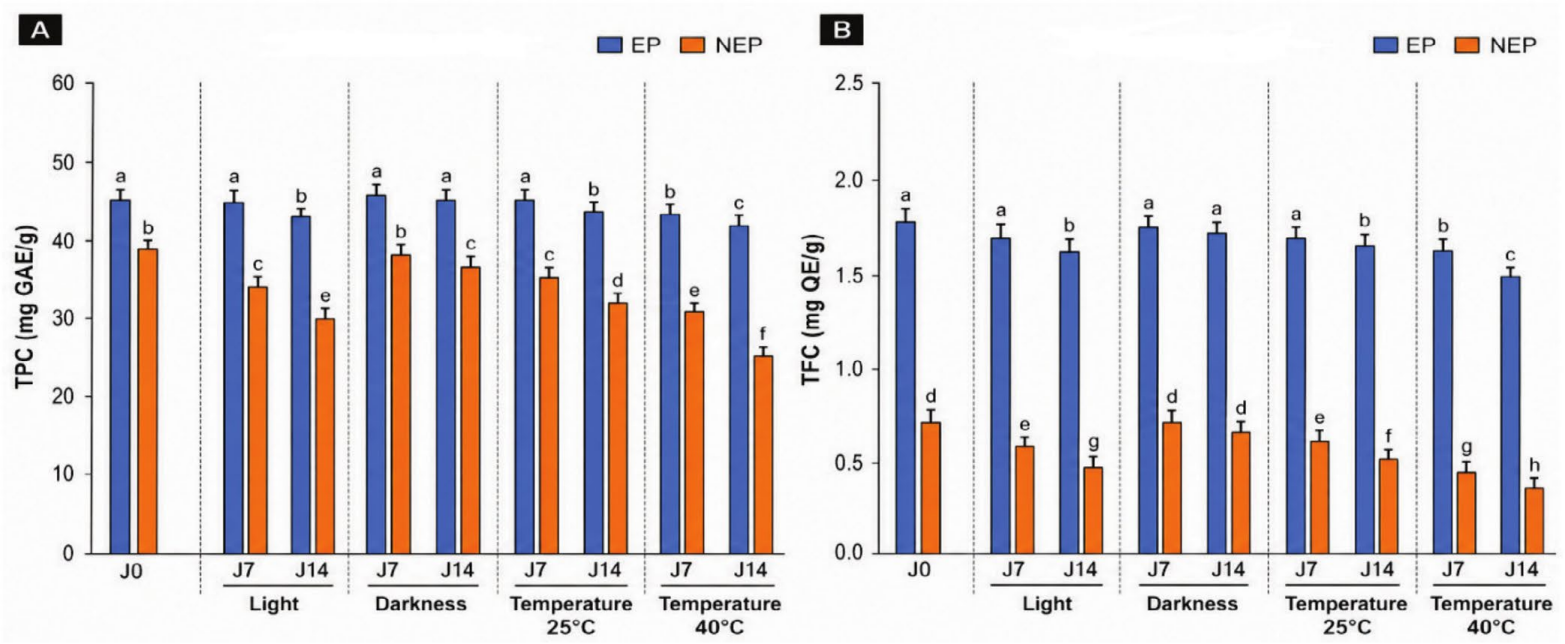
Evaluation of total polyphenols content and total flavonoid content of encapsulated (EP) and non‐encapsulated (NEP) date seed powders under various storage conditions.

#### 3.4.2 Effect of Thermal Stress on Storage Stability

Storage temperature also influenced the retention of the measured bioactive compounds, with greater losses observed at 40°C than at 25°C. This effect was particularly evident for the non‐encapsulated powder. At 25°C, EP retained approximately 96.5% of its initial TPC after 14 days, whereas NEP retained approximately 82.3%. For TFC and TCT, the corresponding retention values were approximately 95.5% and 96.5% for EP, compared with 68.9% and 80.0% for NEP. The differences became more pronounced at 40°C. After 14 days, TPC in EP decreased from 45.81 **±** 0.17 to 41.80 ± 0.40 mg GAE/g, corresponding to approximately 91.3% retention. In contrast, TPC in NEP decreased from 39.01 ± 0.33 to 25.40 ± 0.60 mg GAE/g, corresponding to approximately 65.1% retention. TFC showed a similar pattern, with EP retaining approximately 86.4% of its initial value compared with 51.4% for NEP. TCT retention was approximately 84.7% for EP and 56.6% for NEP, indicating a pronounced loss of condensed tannins in NEP under the higher‐temperature condition (Figure 2). The antioxidant parameters followed the same general pattern. At 40°C and Day 14, EP retained approximately 93.3% of its initial DPPH activity and 88.7% of its ABTS activity, whereas NEP retained approximately 74.9% and 67.9%, respectively. Taken together, these retention profiles indicate that the 40°C storage condition was associated with greater losses of bioactive compounds and antioxidant activity, particularly in the non‐encapsulated powder. The comparatively greater retention observed in EP suggests that encapsulation may have contributed to improving the stability of the bioactive compounds during storage. Similar findings have been reported for spray‐dried phenolic‐rich extracts, for which encapsulation improved the stability of phenolic compounds and antioxidant properties compared with non‐encapsulated systems. In particular, Tolun et al. (2020) demonstrated that the composition of the microencapsulating material played a key role in the storage stability of grape pomace polyphenols, with optimized wall‐material formulations substantially limiting phenolic losses during storage. Likewise, studies on spray‐dried phenolic extracts have shown that the choice of wall material can influence the stability of phenolic compounds and antioxidant activity under different storage conditions (Negrão‐Murakami et al. 2017). The comparatively greater retention observed in EP may therefore be related, at least in part, to the presence of the alginate–xanthan gum wall matrix, which may reduce the direct exposure of the encapsulated compounds to environmental factors during storage. However, this interpretation remains tentative because the physicochemical mechanisms underlying the enhanced stability of EP were not directly investigated in the present study. In particular, parameters such as glass transition temperature, molecular mobility, oxygen permeability, moisture transfer, and matrix microstructure were not characterized. These factors should therefore be regarded as potential contributors rather than experimentally established mechanisms. Overall, the present results demonstrate that encapsulation was associated with substantially higher retention of phenolic compounds, flavonoids, condensed tannins, and antioxidant activity during storage at 40°C. The protective effect was particularly evident for TPC and TCT, for which the difference in retention between EP and NEP was considerable. These findings support the potential of the alginate–xanthan gum encapsulation system to improve the storage stability of date seed bioactive compounds, although further studies under controlled storage conditions and with detailed physicochemical characterization are required to elucidate the mechanisms involved.

**FIGURE 2:**
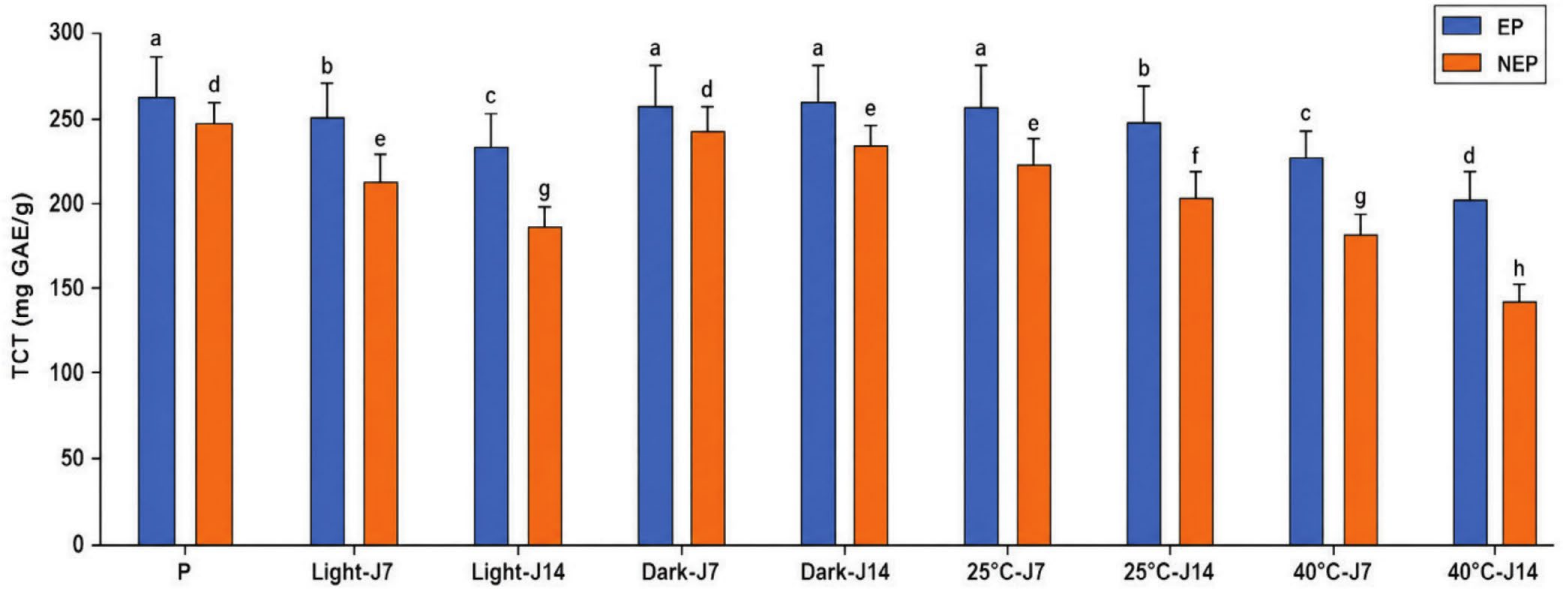
Evaluation of total condensed tannin content of encapsulated (EP) and non‐encapsulated (NEP) date seed powders under various storage conditions.

#### 3.4.3 Relationship Between Bioactive Compound Retention and Antioxidant Activity

The changes observed in the phytochemical parameters were accompanied by corresponding changes in antioxidant activity measured by the DPPH and ABTS assays (Figure 3). The decreases in antioxidant activity were generally more pronounced in NEP, particularly under light exposure and storage at 40°C. At Day 14 and 40°C, for example, DPPH activity decreased to 62.15 ± 0.30 μM Trolox/g in NEP, whereas EP retained a higher value of 78.40 ± 0.12 μM Trolox/g. Similarly, ABTS activity decreased to 68.50 ± 0.45 μM Trolox/g in NEP compared with 92.10 ± 0.20 μM Trolox/g in EP. Under darkness, both powders exhibited comparatively high retention of antioxidant activity. At Day 14, EP retained approximately 99.5% of its initial DPPH activity and 98.9% of its initial ABTS activity, while NEP retained approximately 96.5% and 95.6%, respectively. These observations were consistent with the comparatively high retention of TPC, TFC, and TCT under dark storage. The parallel changes observed between phytochemical retention and antioxidant activity suggest that preservation of phenolic compounds, flavonoids, and condensed tannins may contribute to the maintenance of the antioxidant potential of the powders during storage. However, because the present study did not investigate individual phenolic compounds or establish statistical correlations at the replicate level, these relationships should be interpreted as general associations rather than evidence of a direct causal relationship. The improved stability observed for EP is consistent with previous reports describing the protective effect of encapsulation on phenolic‐rich systems. For example, Kasapoğlu et al. (2022) reported improved stability and bioaccessibility of encapsulated rosehip polyphenols compared with their unencapsulated counterparts. The present results similarly indicate that encapsulation can contribute to the preservation of antioxidant potential during exposure to environmental storage stresses.

**FIGURE 3:**
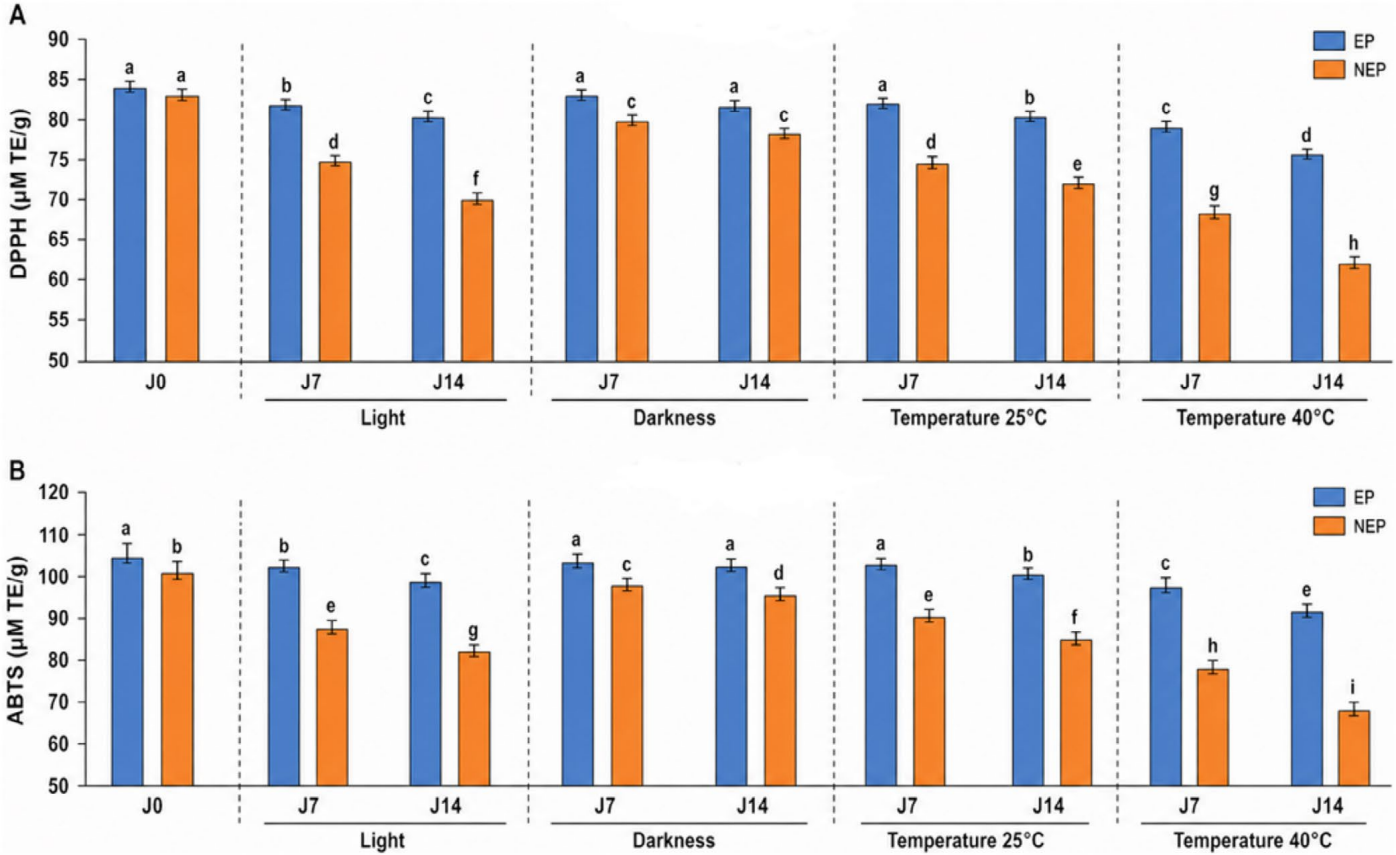
Evaluation of antioxidant activity of encapsulated (EP) and non‐encapsulated (NEP) date seed powders under various storage conditions.

The storage results demonstrate a consistent descriptive pattern of greater bioactive compound and antioxidant retention in EP than in NEP (Table 4). Darkness represented the most favorable storage condition, whereas storage at 40°C resulted in the greatest losses, particularly in NEP. At Day 14, EP retained 91.3%–93.3% of its initial TPC and DPPH activity at 40°C, while NEP retained 65.1%–74.9%, respectively. Under light exposure, the corresponding retention of TPC and DPPH was approximately 95.0% and 96.6% for EP compared with 77.3% and 84.8% for NEP. These findings support the potential of the alginate–xanthan gum encapsulation system to improve the storage stability of date seed bioactives. Nevertheless, the storage experiment was limited to 14 days and the investigated environmental conditions. In addition, the individual replicate‐level observations required for a new inferential statistical analysis were not available at the time of revision. Therefore, the present storage findings are interpreted descriptively and no new statistical significance or interaction effects are inferred from the aggregated mean ± SD values. Longer‐term studies conducted under controlled temperature, humidity, light, and oxygen conditions, combined with characterization of the physicochemical properties of the encapsulation matrix, would be valuable for establishing the mechanisms governing stability and for determining the practical shelf‐life of the encapsulated date seed powder.

**TABLE 4:** Storage stability of bioactive compounds and antioxidant activity of encapsulated (EP) and non‐encapsulated (NEP) date seed powders.

Storage condition	Day	Sample	TPC (mg GAE/g)	TFC (mg QE/g)	TCT (mg CE/g)	DPPH (μM Trolox/g)	ABTS (μM Trolox/g)
Initial	J0	EP	45.81 ± 0.17	1.76 ± 0.01	360.85 ± 0.21	84.05 ± 0.01	103.81 ± 0.01
Initial	J0	NEP	39.01 ± 0.33	0.74 ± 0.01	256.68 ± 0.44	83.02 ± 0.02	100.83 ± 0.01
Light	J7	EP	44.80 ± 0.20	1.70 ± 0.02	350.20 ± 0.40	82.80 ± 0.05	101.50 ± 0.10
Light	J7	NEP	34.20 ± 0.40	0.58 ± 0.02	215.10 ± 0.60	75.40 ± 0.15	88.20 ± 0.20
Light	J14	EP	43.50 ± 0.25	1.65 ± 0.03	335.10 ± 0.50	81.20 ± 0.08	98.50 ± 0.15
Light	J14	NEP	30.15 ± 0.50	0.48 ± 0.03	190.40 ± 0.70	70.40 ± 0.20	82.30 ± 0.25
Darkness	J7	EP	45.50 ± 0.15	1.74 ± 0.01	358.40 ± 0.30	83.80 ± 0.03	103.10 ± 0.05
Darkness	J7	NEP	37.90 ± 0.30	0.71 ± 0.01	248.50 ± 0.45	81.50 ± 0.10	98.10 ± 0.12
Darkness	J14	EP	45.10 ± 0.18	1.73 ± 0.02	355.80 ± 0.35	83.60 ± 0.05	102.70 ± 0.08
Darkness	J14	NEP	36.80 ± 0.35	0.68 ± 0.02	240.15 ± 0.50	80.15 ± 0.12	96.40 ± 0.15
25°C	J7	EP	45.20 ± 0.22	1.72 ± 0.01	355.60 ± 0.40	83.40 ± 0.04	102.60 ± 0.09
25°C	J7	NEP	35.40 ± 0.38	0.62 ± 0.02	230.15 ± 0.55	77.80 ± 0.18	91.40 ± 0.22
25°C	J14	EP	44.20 ± 0.28	1.68 ± 0.02	348.15 ± 0.45	82.80 ± 0.06	100.95 ± 0.12
25°C	J14	NEP	32.10 ± 0.45	0.51 ± 0.03	205.40 ± 0.65	72.50 ± 0.22	85.60 ± 0.30
40°C	J7	EP	43.60 ± 0.30	1.64 ± 0.03	332.40 ± 0.60	81.50 ± 0.08	97.40 ± 0.15
40°C	J7	NEP	31.20 ± 0.55	0.45 ± 0.04	185.30 ± 0.80	68.40 ± 0.25	78.15 ± 0.35
40°C	J14	EP	41.80 ± 0.40	1.52 ± 0.04	305.60 ± 0.75	78.40 ± 0.12	92.10 ± 0.20
40°C	J14	NEP	25.40 ± 0.60	0.38 ± 0.05	145.20 ± 0.95	62.15 ± 0.30	68.50 ± 0.45

### 3.5 Evolution of Bioactive Compounds During Simulated Gastrointestinal Digestion (SGID)

It is widely recognized that phenolic compounds are the main bioactive constituents responsible for the health‐promoting effects of plant‐derived foods and their co‐products (Ranasinghe et al. 2025).

However, their measurable concentration during gastrointestinal digestion depends not only on their initial abundance but also on their release from the food matrix, their chemical stability under changing gastrointestinal conditions, and their interactions with other components of the digestive system. In the present study, the evolution of total phenolic content (TPC), total flavonoid content (TFC), and total condensed tannin content (TCT) was monitored during the oral, gastric, and intestinal phases of the INFOGEST 2.0 in vitro digestion model (Table 5).

**TABLE 5:** Total phenolic content (TPC), total flavonoid content (TFC), total condensed tannin content (TCT), and bioaccessibility index (BI) of encapsulated (EP) and non‐encapsulated (NEP) date‐seed powders during the oral, gastric, and intestinal phases of the INFOGEST 2.0 in vitro digestion model. Values are expressed as mean ± SD (*n* = 3).

Parameter	Sample	Oral phase	Gastric phase	Intestinal phase	Bioaccessibility index (BI, %)
TPC (mg GAE/g DM)	EP	43.52 ± 0.22	39.18 ± 0.19	30.24 ± 0.25	66
	NEP	36.10 ± 0.31	31.25 ± 0.28	22.63 ± 0.30	58
TFC (mg QE/g DM)	EP	1.672 ± 0.006	1.514 ± 0.005	1.162 ± 0.007	—
	NEP	0.690 ± 0.002	0.596 ± 0.002	0.432 ± 0.002	—
TCT (mg CE/g DM)	EP	342.81 ± 0.33	309.33 ± 0.29	238.16 ± 0.35	—
	NEP	237.43 ± 0.50	205.35 ± 0.47	148.88 ± 0.48	—

*Note:* BI (%) = [TPC at the intestinal phase/TPC before simulated gastrointestinal digestion] × 100. The BI values were calculated using the TPC measured before digestion as the initial reference value. BI values are presented as calculated percentages; statistical comparison between EP and NEP based on the independent replicate‐level BI values.

#### 3.5.1 Oral Phase

Exposure to the simulated oral phase resulted in a modest reduction in the measured concentrations of the evaluated phytochemical groups in both powders (Table 5). For the encapsulated powder (EP), TPC, TFC, and TCT reached 43.52 ± 0.22 mg GAE/g DM, 1.672 ± 0.006 mg QE/g DM, and 342.81 ± 0.33 mg CE/g DM, respectively. The corresponding values for the non‐encapsulated powder (NEP) were 36.10 ± 0.31 mg GAE/g DM, 0.690 ± 0.002 mg QE/g DM, and 237.43 ± 0.50 mg CE/g DM. The relatively limited changes observed during this short digestion stage may reflect the brief exposure time and the physicochemical conditions of the simulated oral phase. The higher measured concentrations in EP compared with NEP indicate that encapsulation was associated with greater retention of the investigated phytochemical compounds during the initial digestion step. This observation is consistent with the potential role of the alginate–xanthan matrix in limiting the immediate exposure of the extract to the digestive medium. The decrease in measurable phenolic compounds may also be related to interactions between phenolic constituents and components of the simulated oral environment. Phenolic compounds, particularly tannins, can interact with proteins through non‐covalent interactions such as hydrogen bonding and hydrophobic interactions, potentially modifying their solubility and their recovery in analytical assays (Czubinski and Dwiecki 2017; Wang et al. 2022). However, the present study did not directly characterize these molecular interactions. Therefore, the observed decrease should be interpreted as a change in measurable phenolic content rather than as direct evidence of enzymatic degradation or formation of specific phenolic–protein complexes.

#### 3.5.2 Gastric Phase

A further decrease in TPC, TFC, and TCT was observed during the gastric phase, which was conducted under acidic conditions in the presence of pepsin (Table 5). For NEP, TPC decreased to 31.25 ± 0.28 mg GAE/g DM, while TFC and TCT decreased to 0.596 ± 0.002 mg QE/g DM and 205.35 ± 0.47 mg CE/g DM, respectively. In EP, the corresponding values were 39.18 ± 0.19 mg GAE/g DM for TPC, 1.514 ± 0.005 mg QE/g DM for TFC, and 309.33 ± 0.29 mg CE/g DM for TCT. Compared with NEP, EP retained higher levels of all three phytochemical indicators following gastric digestion. This difference suggests that encapsulation was associated with greater retention of the measured phytochemical constituents under the investigated gastric conditions. The higher retention observed for EP may be associated with the protective properties of the alginate–xanthan gum matrix and its potential influence on the exposure and release of encapsulated compounds. However, matrix integrity and compound‐release behavior were not directly characterized in the present study; therefore, the specific mechanism underlying the higher retention cannot be confirmed. The reduction in measurable TPC, TFC, and TCT during the gastric phase may reflect several processes, including changes in compound solubility, interactions with digestive components, and chemical transformation of individual phenolic constituents under acidic conditions. Conversely, acidic digestion may also promote the release of compounds that were initially associated with the powder matrix. Thus, changes in global TPC, TFC, and TCT should not be interpreted exclusively as evidence of compound degradation. The higher levels of TPC, TFC, and TCT retained by EP after gastric digestion indicate that encapsulation influenced the fate of the measured phytochemical constituents during simulated gastrointestinal digestion. However, the present results do not establish a specific release or degradation mechanism, and further studies incorporating matrix characterization and compound‐specific analytical approaches would be required to elucidate the underlying processes (Brodkorb et al. 2019).

#### 3.5.3 Intestinal Phase and Bioaccessibility (% BI)

The intestinal phase resulted in the lowest measured concentrations of TPC, TFC, and TCT for both formulations (Table 5). In NEP, TPC decreased to 22.63 ± 0.30 mg GAE/g DM and TFC to 0.432 ± 0.002 mg QE/g DM, while TCT reached 148.88 ± 0.48 mg CE/g DM. In comparison, EP retained higher concentrations, with TPC, TFC, and TCT values of 30.24 ± 0.25 mg GAE/g DM, 1.162 ± 0.007 mg QE/g DM, and 238.16 ± 0.35 mg CE/g DM, respectively. The higher concentrations measured for EP during the intestinal phase indicate greater retention of the evaluated phytochemical constituents following simulated gastrointestinal digestion. The higher retention observed for EP may reflect differences in compound release and stability associated with the encapsulation matrix. However, the specific molecular mechanisms underlying this behavior were not determined in the present study.

The intestinal bioaccessibility index (BI) was calculated from TPC according to the following equation:

$$\mathrm{BI}\;\left(\%\right)=\left(\mathrm{TPC}\;\text{intestinal phase}/\mathrm{TPC}\;\text{initial}\right)\times 100$$

Based on the calculated values, the TPC‐based BI was approximately 66% for EP and 58% for NEP. These values indicate that a greater proportion of the initially measured phenolic content remained measurable after the intestinal digestion stage in the encapsulated formulation. However, the difference between the two formulations should be interpreted statistically only on the basis of independent replicate‐level BI values. Accordingly, the BI values are reported descriptively in Table 6, and no claim of a statistically significant difference between EP and NEP should be made unless supported by an appropriate statistical comparison using the independent experimental replicates. The decrease in measurable phenolic content observed throughout digestion differs from reports in which intestinal digestion resulted in increased concentrations of phenolic compounds or phenolic acids (Tian et al. 2021). Such increases have been associated with the release of matrix‐bound compounds, hydrolysis of conjugated forms, or enhanced extractability under intestinal conditions. The contrasting behavior observed in the present study may reflect differences in the composition of date‐seed biomass, the chemical characteristics of its phenolic fraction, and the experimental digestion conditions. In addition, the use of global spectrophotometric assays provides an estimate of the total measurable phenolic, flavonoid, or tannin fraction rather than the concentration of individual compounds. Consequently, changes in TPC, TFC, and TCT cannot be directly interpreted as evidence of the degradation or formation of specific phenolic molecules. Overall, the SGID results indicate that encapsulation was associated with greater retention of the measured phytochemical constituents throughout the simulated gastrointestinal process and with a higher TPC‐based intestinal bioaccessibility index than the non‐encapsulated formulation. Nevertheless, the INFOGEST 2.0 model provides an estimate of in vitro bioaccessibility and does not directly measure intestinal absorption, systemic bioavailability, metabolism, or biological effects in vivo. Further studies incorporating detailed chromatographic profiling and appropriate intestinal absorption models would therefore be required to characterize the fate and potential bioavailability of individual date‐seed phenolic compounds (de Paulo Farias et al. 2021).

**TABLE 6:** DPPH and ABTS antioxidant activities of encapsulated (EP) and non‐encapsulated (NEP) date‐seed powders during the oral, gastric, and intestinal phases of the INFOGEST 2.0 in vitro digestion model. Values are expressed as mean ± SD (*n* = 3).

Digestion phases	Samples	DPPH (μM Trolox/g)	ABTS (μM Trolox/g)
Oral phase	EP	79.85 ± 0.02	98.62 ± 0.01
	NEP	74.71 ± 0.03	90.75 ± 0.04
Gastric phase	EP	71.44 ± 0.01	88.24 ± 0.02
	NEP	58.11 ± 0.05	70.58 ± 0.03
Intestinal phase	EP	55.47 ± 0.03	68.51 ± 0.02
	NEP	48.15 ± 0.04	58.48 ± 0.05

### 3.6 Impact of In Vitro Digestion on Antioxidant Capacity

The antioxidant activity of the encapsulated (EP) and non‐encapsulated (NEP) powders, evaluated using the DPPH and ABTS assays, decreased progressively across the simulated gastrointestinal digestion phases (Table 6). This overall trend was broadly consistent with the reductions in TPC, TFC, and TCT observed during digestion. Nevertheless, EP generally retained higher antioxidant activity than NEP at each digestion stage. During the oral phase, DPPH activity was 79.85 ± 0.02 μM Trolox/g for EP and 74.71 ± 0.03 μM Trolox/g for NEP, while ABTS activity reached 98.62 ± 0.01 and 90.75 ± 0.04 μM Trolox/g, respectively. Following the gastric phase, DPPH activity decreased to 71.44 ± 0.01 μM Trolox/g in EP and 58.11 ± 0.05 μM Trolox/g in NEP. Similarly, ABTS activity decreased to 88.24 ± 0.02 μM Trolox/g for EP and 70.58 ± 0.03 μM Trolox/g for NEP. These results indicate greater retention of antioxidant activity in the encapsulated formulation under the investigated gastric conditions.

The intestinal phase resulted in a further decrease in radical‐scavenging activity in both formulations. DPPH activity reached 55.47 ± 0.03 μM Trolox/g for EP compared with 48.15 ± 0.04 μM Trolox/g for NEP, whereas ABTS activity decreased to 68.51 ± 0.02 and 58.48 ± 0.05 μM Trolox/g, respectively. Thus, although both formulations showed reduced antioxidant activity following gastrointestinal digestion, EP retained higher DPPH and ABTS activities than NEP at the intestinal stage.

The progressive changes in antioxidant activity were broadly consistent with the evolution of the global phytochemical indicators reported in Table 5. Phenolic compounds, flavonoids, and condensed tannins can contribute to radical‐scavenging activity; therefore, changes in their measurable concentrations may be associated with changes in DPPH and ABTS responses. However, the present study did not determine the contribution of individual phenolic compounds to antioxidant activity. Accordingly, the observed relationships should be interpreted as overall associations rather than evidence of direct causal relationships.

The higher antioxidant activity retained by EP under the investigated gastric and intestinal conditions may be associated with the protective properties of the alginate–xanthan gum matrix. Encapsulation may modify the exposure of extract constituents to the digestive environment and consequently influence their retention and measurable antioxidant activity. However, direct degradation of antioxidant compounds by the acidic environment, digestive enzymes, or other components of the simulated gastrointestinal media was not investigated in the present study. Similarly, matrix integrity, compound‐release behavior, and specific molecular interactions were not directly characterized. Therefore, the precise mechanisms underlying the greater retention observed in EP cannot be established from the present data.

Overall, the results indicate that encapsulation was associated with greater retention of DPPH‐ and ABTS‐scavenging activity during the simulated gastrointestinal digestion process. These findings suggest that the alginate–xanthan gum matrix may contribute to the retention of antioxidant activity under the investigated in vitro digestion conditions. However, the antioxidant assays used in this study provide chemical measures of radical‐scavenging capacity and should not be interpreted as direct evidence of in vivo antioxidant effects or health benefits. Further studies combining compound‐specific chromatographic profiling, matrix characterization, and intestinal absorption models would be necessary to elucidate the fate and biological relevance of the individual antioxidant constituents.

## 4 Conclusion

The present study demonstrates the potential of Tunisian date seeds as an underutilized source of valuable bioactive compounds and supports their valorization within a circular and sustainable food‐processing framework. Among the extraction strategies investigated, aqueous high‐pressure extraction provided the most favorable recovery of phenolic compounds and antioxidant activity under the specific experimental conditions employed. The selected extract was subsequently incorporated into an alginate–xanthan gum matrix by spray drying, and the resulting encapsulated powder showed improved retention of measured bioactive fractions during storage compared with the non‐encapsulated powder. This improved retention may be associated with the protective properties of the alginate–xanthan gum matrix; however, the specific physicochemical mechanisms responsible for this effect were not directly investigated. The simulated gastrointestinal digestion experiments further indicated that encapsulation was associated with greater retention of the measured phenolic, flavonoid, tannin, and antioxidant fractions under the investigated in vitro conditions. The TPC‐based intestinal bioaccessibility index was approximately 66% for the encapsulated powder compared with 58% for the non‐encapsulated powder. These findings support the potential of integrating extraction and stabilization steps to obtain date‐seed‐derived ingredients with improved retention of measurable bioactive constituents. However, the results should be interpreted within the scope of the experimental conditions and the in vitro nature of the study and should not be extrapolated to intestinal absorption, in vivo bioavailability, or health effects.

Several limitations should be considered. First, the study was conducted at laboratory scale and storage stability was evaluated for only 14 days; consequently, long‐term shelf‐life behavior and performance under industrial storage conditions remain to be established. Second, phytochemical characterization relied primarily on global spectrophotometric indicators, including TPC, TFC, TCT, DPPH, and ABTS, and did not include compound‐level identification or quantification by HPLC‐DAD, LC–MS, or comparable chromatographic techniques. Therefore, the present findings cannot establish the behavior of individual phenolic compounds during extraction, storage, or gastrointestinal digestion. Third, the extraction treatments were not evaluated using a full factorial UAE × HPAE design. Consequently, the sequential UAE–HPAE treatment cannot be used to establish synergistic, additive, or antagonistic interactions between the two extraction approaches and should be interpreted only as a specific sequential treatment condition. Fourth, the encapsulation efficiency was moderate, indicating that further optimization of wall‐material composition, feed formulation, and spray‐drying parameters is warranted. Moreover, morphological and quantitative particle‐size characterization was not performed. Finally, although the INFOGEST 2.0 model provides valuable information on in vitro gastrointestinal bioaccessibility, it does not reproduce the complexity of intestinal absorption, metabolism, systemic bioavailability, or biological responses in vivo.

Future research should therefore focus on compound‐specific phytochemical characterization using chromatographic and mass‐spectrometric approaches, together with systematic optimization of extraction and spray‐drying parameters. Longer‐term storage studies under controlled temperature, humidity, and light conditions should be conducted, complemented by morphological and particle‐size characterization of the powders. Further work should also assess process energy requirements, material efficiency, economic feasibility, and scale‐up performance to determine the practical potential of the proposed valorization approach. Appropriate intestinal absorption models and in vivo studies will additionally be required to determine whether the observed in vitro bioaccessibility is associated with enhanced intestinal uptake or biological effects.

Overall, this study provides a laboratory‐scale basis for the integrated valorization of Tunisian date‐seed residues through aqueous high‐pressure extraction and spray‐drying encapsulation. The findings support the potential of this approach for producing bioactive‐rich powders with improved retention under the investigated storage and simulated gastrointestinal conditions. Nevertheless, further compositional, technological, storage, economic, and biological validation is required before the approach can be considered for broader food or nutraceutical applications.

## Author Contributions

**Olfa Rebai:** conceptualization, investigation, writing – original draft, methodology, writing – review and editing, resources. **Faten Raboudi:** formal analysis, resources. **Malek Ben Temessek:** software, resources, methodology. **Rafik Gatri:** writing – review and editing, software. **Sami Fattouch:** conceptualization, project administration, supervision.

## Funding

This work was supported by Partnership for Research and Innovation in the Mediterranean Area (2132).

## Ethics Statement

The authors have nothing to report.

## Conflicts of Interest

The authors declare no conflicts of interest.

## Data Availability

The data that support the findings of this study are available on request from the corresponding author. The data are not publicly available due to privacy or ethical restrictions.
